# Interventions to Reduce Adult Nursing Turnover: A Systematic Review of Systematic Reviews

**DOI:** 10.2174/1874434601711010108

**Published:** 2017-08-15

**Authors:** Mary Halter, Ferruccio Pelone, Olga Boiko, Carole Beighton, Ruth Harris, Julia Gale, Stephen Gourlay, Vari Drennan

**Affiliations:** 1 Faculty of Health, Social Care and Education, Kingston University and St George’s, University of London, London, England; 2 National Guideline Alliance, Royal College of Obstetricians and Gynaecologists, London, England; 3 Nursing & Midwifery, King's College London, England; 4 Faculty of Business & Law, Kingston University, London, England

**Keywords:** Intervention, Nurses, Nursing staff, Personnel turnover, Review, Systematic, Workforce

## Abstract

**Background:**

Nurse turnover is an issue of concern in health care systems internationally. Understanding which interventions are effective to reduce turnover rates is important to managers and health care organisations. Despite a plethora of reviews of such interventions, strength of evidence is hard to determine.

**Objective:**

We aimed to review literature on interventions to reduce turnover in nurses working in the adult health care services in developed economies.

**Method:**

We conducted an overview (systematic review of systematic reviews) using the Cochrane Database of Systematic Reviews, MEDLINE, EMBASE, Applied Social Sciences Index and Abstracts, CINAHL plus and SCOPUS and forward searching. We included reviews published between 1990 and January 2015 in English. We carried out parallel blinded selection, extraction of data and assessment of bias, using the Assessment of Multiple Systematic Reviews. We carried out a narrative synthesis.

**Results:**

Despite the large body of published reviews, only seven reviews met the inclusion criteria. These provide moderate quality review evidence, albeit from poorly controlled primary studies. They provide evidence of effect of a small number of interventions which decrease turnover or increase retention of nurses, these being preceptorship of new graduates and leadership for group cohesion.

**Conclusion:**

We highlight that a large body of reviews does not equate with a large body of high quality evidence. Agreement as to the measures and terminology to be used together with well-designed, funded primary research to provide robust evidence for nurse and human resource managers to base their nurse retention strategies on is urgently required.

## 1 INTRODUCTION

Turnover amongst nurses, that is, nurses leaving their jobs or leaving the profession, has received international attention due to the size of the issue [1] and the consequences of high rates of turnover [2]. Supply demand gaps are projected in developed economies such as Australia [3], Canada [4], the United States of America [5] and in the United Kingdom (UK) [6], as are shortages in many other EU countries [7]. Negative consequences of such shortages for the nursing workforce in less developed economies is well documented [7]. Nurse turnover is described as having a large number of individual, organisational and societal antecedents or determinants and, although the strength of the evidence is not as strong as its size suggests, a number of causal or correlational models from nursing exist and have some overlap with those from the broader human resource management literature [8]. A pressing issue for nurse and human resource managers in developed economies is to introduce interventions which are effective in addressing those determinants, reducing nurse turnover and increasing nurse retention [9]. A series of policy initiatives (in the UK National Health Service) were put in place in the early 2000s [10] but it has been suggested that ‘it is unclear how effective the initiatives were in retaining staff and whether they were fully implemented.’ [11,p16] A recent review aiming to support the growth of nurse numbers concluded that adopting a number of strategies, including some based on evaluated intervention (for example Gess 2008 [12] or Hirschkorn 2010 [13]), although the review did not appraise the quality of these studies [10].

Outside of nursing, the human resources literature on retention management is reported to describe a large number of practices and groups of practice that have been shown to be effective in the retention of individuals, although a conceptualisation of them as a whole has not been forthcoming [14]. Reported strategies include performance-based reward systems, long-term career prospects, personal recruitment and socialisation all based on retaining an individual although a model for the retention of resource has also been proposed as an alternative way of considering the issue of retention [14].

Our awareness of the existence of some literature focused on nursing retention alongside the human resource literature based on many different job roles and settings, led us to undertake a preliminary stage of review - making an assessment of potentially relevant literature specific to nursing and its size for review [15] - when we were commissioned to carry out a review of the adult nurse turnover literature. Using Medline alone at this stage we identified a large body of reviews (Table **1**) relevant to the study’s objectives that indicated that nurse and human resource managers would be faced by a plethora of reviews of interventions to reduce turnover in adult nursing [16, 17], many of which were not conducted according to reviews guidance [15].

Against this background, the aim of this research was to undertake an overview (that is a systematic review of systematic reviews [18]) on interventions to reduce turnover in nurses working in the field of adult health care services, that is the largest group of nurses in all countries [19-21]. Our desire to provide a review that might be used by nurses in practice informed our decision to use an overview, whose purpose “in identifying and appraising all published reviews is to describe their quality, summarise and compare their conclusions and discuss the strength of these conclusions, so that the best evidence is made available to clinical decision-makers.” [22].

## 2 MATERIAL AND METHODS

The review methods and the reporting of them are based on the guideline from the Preferred Reporting Items for Systematic Review and Meta-analysis Protocols (PRISMA-P) 2015 statement [23] and the guidance within the Cochrane Handbook for Systematic Reviews of Interventions [18, 24].

### 2.1 Criteria for Inclusion

The inclusion criteria were as follows:

- Published from 1990 onwards
- Population: The review was focused on those delivering adult nursing (*i.e.* licensed or registered) in health services (both in hospital and community) in developed economies (according to the definition of the International Monetary Fund [25])
- Intervention: The review examined any type of service, management or human resources activity aimed at reducing rates of adult nurse turnover
- Comparison: Any comparators used within the included reviews
- Outcomes: Any outcome examined within the included reviews
- Review design: Any form of literature review which had been peer-reviewed, contained a statement of review, reported its search strategy and/or inclusion/exclusion criteria, reported either empirical findings or a list of included primary studies and included a methodological quality assessment of its included primary studies, that is a review containing key aspects of a well-conducted systematic review

Exclusion criteria were as follows: Reports from any types of primary studies; reviews published in language other than English; reviews that did not evaluate adult nursing turnover as described in the inclusion criteria or presented data on nurses working across settings that could include the care of children or in specific mental health settings; reviews that did not report empirical findings; reviews published only in abstract form; any form of literature review using informal and subjective methods to collect and interpret evidence; commentaries and non peer-reviewed reviews; any review in which the majority of included articles were non-peer reviewed publications and reviews that did not report an appraisal of the quality of the studies they included.

### 2.2 Search Methods for Identification of Studies

We searched the Cochrane Database of Systematic Reviews, MEDLINE (Ovid), EMBASE (Ovid), Applied Social Sciences Index and Abstracts ASSIA, CINAHL plus (EBSCO) and SCOPUS V.4 (Elsevier) from 1990 to 2015 (searches conducted January 2015). Search strategies were guided by a systematic approach to the research questions [26] and a Medline search strategy was developed (Table **1**) and converted or modified to run on other databases **(Supplementary file 1)**. We identified additional studies by searching on PubMed by using the “related citations” algorithm and screening the reference lists of included studies on for other reviews [27].

### 2.3 Selection of Studies

The results of the electronic search were downloaded into an Excel spreadsheet After removing duplicate articles, relevant reviews were selected according to eligibility criteria using a two-step screening process:

- Title and abstract screening: Two authors (FP and MH) reviewed in parallel the titles and abstracts of all the articles resulted to ascertain their eligibility for full text retrieval. Disagreements were resolved by peer discussion and a third view from the project lead (VMD) if required.
- Full-text screening: Two reviewers (FP and CB) read in parallel all the selected full-text articles to analyse whether they met the inclusion/exclusion criteria. Any discrepancies between the two reviewers were resolved in discussion with the third reviewer (MH).

### 2.4 Data Extraction

Data were extracted to excel spreadsheets (FP and CB) using a predefined extraction form and spreadsheet on: *general characteristics of the review* *e.g.* author(s), year, geographical scope, research area, and authors’ aims/ research question(s); *descriptive characteristics* *e.g.* type of review (design), selection criteria to include primary studies, number and study designs of articles incorporated in the reviews and outcome measures; *results-* effectiveness of all interventions reported in the included reviews, the direction of findings against the outcome measure and the references for the primary studies; *main conclusions,* using the review authors’ words and *limitations*, as noted by the review authors. Discrepancies were resolved through discussion among the data extractors.

The primary studies included in each review were also listed and compared across the reviews to assess the degree of overlap in the reviews we included.

### 2.5 Assessment of Methodological Quality

The 11-point Assessment of Multiple Systematic Reviews (AMSTAR) checklist [28] was used to assess the quality of each included review. This tool has been widely used in previous similar overview of reviews and it is considered to be a valid and reliable instrument [29]. Using the AMSTAR scale two authors appraised each included paper. Any review that scored eight or higher was considered at low risk of bias, between five and seven at moderate risk of bias and four or less at high risk of bias.

### 2.6 Data Analysis

Because of the heterogeneous nature of the focus, inclusion criteria and outcome measures of the included studies data were analysed thematically. Following the detailed reading involved for data extraction, the resultant spreadsheet was examined and a thematic index of interventions developed. The thematic index **(supplementary data file 2)** was applied to each data extraction and four main groupings of interventions (individual, leadership, group and organisational levels) were used to analyse across reviews, using Microsoft Excel 2010 to record the decisions applied for all reviews considered. A narrative account of the findings from the reviews containing an assessment of the methodological quality of included primary studies has been structured using the risk of bias in the review as the primary grouping level and the thematic content analysis as the second level, also drawing on the number and quality of the included primary studies. In this way we aim to describe the findings by ‘weight of evidence’. [30]

The systematic review protocol was registered with PROSPERO 2015: CRD42015017535 [31].

## 3 RESULTS: REVIEW SELECTION, STUDY CHARACTERISTICS AND QUALITY ASSESSMENT

### 3.1 Review Selection

The flow chart representing study selection, including reasons for exclusion, is summarised in Fig. (**1**). A total of seven reviews met the inclusion criteria and were included in the review. **Supplementary File 3** provides a list of citations for the excluded studies in the final stage of the selection process.

### 3.2 Study Characteristics

Details of the seven included reviews are provided in Tables (**2**, **3** and **4**). The included reviews were conducted between 2008 and 2014. All were published in English and originated from Canada [32-35], the United States [36, 37] and Taiwan [38]. Five reviews focused on the effectiveness of retention strategies targeted at registered nurses [33, 38] or newly graduated nurses [34-36]. One review aimed to examine the relationship between managers’ leadership practices and staff nurses’ intent to stay in their current position [32], and another focused on a single intervention: sabbaticals as strategy to enhance nursing retention and revitalisation to generate positive outcomes [37].

### 3.3 Quality Assessment of Included Reviews

The AMSTAR scores for the reviews ranged from three to seven. Of the reviews, six [32-36, 38] were judged of moderate quality and one [37] of low quality. The assessment of each review against the AMSTAR criteria is presented in Fig. (**2**).

All reviews noted that the methods of the included studies were different, and that overall quality of the primary study ranged from high to low. The tools used to assess the quality of included papers in the included studies are shown in Table **3**.

The majority of the reviews limited their searches to the English language, with the exception of two reviews [33, 36]. The number of included primary studies in each review ranged from five to forty-seven Table (**3**). Three systematic reviews included quasi-experimental study designs [33, 34, 38]; however, observational study designs dominated, and no review reported any randomised controlled trials. Only two reviews included qualitative or mix-methods primary studies [32, 34]. Of the 164 primary studies in the seven included reviews, 14 were included in at least two reviews, and of these only four papers of primary studies [39-42] were included in three reviews Table (**4**).

## 3.4 Findings on Interventions to Reduce Turnover in Adult Nursing

The evidence from the included reviews is presented here narratively by thematic analysis of interventions, grouped into four content categories: individual, job-related, interpersonal, and organisational interventions; unless otherwise stated, the reviews were of moderate quality. In addition Tables (**3** and **4**) split the presentation of the supporting detail of these reviews by the type of interventions reviewed.

### 3.5 Interventions at the Individual Level

At the individual level, interventions were heavily but not exclusively focused on newly qualified/graduated nurses (NGNs), and on supportive programmes of transition or development. Preceptorship - one-to-one guidance through clinical experience - was a component of the majority, alongside a range of programme components (for example, classroom learning or group discussion) and support systems (for example, the programme director or clinical educator). These programmes were variously named, including the terms residency, internship and orientation as well as mentoring and preceptorship itself.

Residency received positive support in four reviews [34-36, 38] reporting several studies, with some overlapping reviews. One of these reviews did not provide the description of the study designs, although the authors noted that the excluded studies appraised to be of low quality [36], and another described its three included studies as one pre test-post test and two experimental case study designs [35]. The included studies measure the turnover outcome in the experimental group and compare it to general pre-published reports locally or nationally.

Internships also received some emphasis as positive for retention, supported in two reviews [35, 36], drawing on six studies of variable design but including one with a controlled pre test-post test design. Orientation focus programmes for transition were highlighted in three reviews [34-36] as positively impacting on turnover in four primary studies.

One to one mentorship programmes of three months’ duration were reported as essential to retaining newly registered nurses, reducing turnover in two quasi experimental pretest-posttest studies (from the USA and Taiwan) in one review [38]. For preceptorship itself there was evidence in one review [35], from ten studies from the USA, all of which were experimental in design, although six were one group case studies, two were one group pretest-posttest and only two included a control group (nonrandomised) pretest-posttest design. Half of these studies were also reported in the review to have included a focus on supporting RNs to work in the preceptor capacity through educational training and/or monetary incentives [35].

Evidence for the positive impact of externships (preceptored and employment experiences of the student nurse the year before graduation from a basic RN education programme) was limited to one study reported in one review [35]. Needs-based training or specialty training programmes (designed to develop skills for specific clinical areas and including classroom instruction, observational experience, journaling, case study, coaching, and computer-based training) was also reported to increase retention, although only one of the primary studies (where the design was clearly reported by the review authors [35]) was robust, with a control arm.

These reviews vary in how actual turnover rates are reported, making comparison or synthesis difficult. For example, while the rates of turnover compare favourably with average ranges for turnover or retention of new graduates, “few studies had designs with the degree of control necessary to rule out competing explanations”[34]. With the same caveats about strength of evidence, longer transition programmes (up to one year) appeared to achieve better results regarding turnover [34, 35].

Other interventions identified at the individual level in the moderate quality reviews were bicultural training which was positively associated with turnover in one study in one review [35], and degree of fit to the job/lower work abilities which was negatively associated with turnover in one study in another review [33].

In addition, one review of poor quality/high risk of bias [37], suggested that a clinical practice sabbatical (a leave of absence for an identified purpose) for nurses in acute care settings was a viable option as a strategy to increase retention. Descriptions of the 19 included articles in this review are unclear, but it appears that only five addressed nursing sabbaticals and retention and all were at best descriptive studies and at worst anecdotal accounts; the review authors conclude that the evidence is limited.

### 3.6 Interventions at the Leadership Level

Two reviews addressed interventions at the leadership level. Management training in leadership behaviour featured in one study in one review [33], and supervision support in seven primary studies in another review [32], as significantly related to intent to stay.

### 3.7 Interventions at the Organisational Level

Two reviews considered interventions at the group or organisational level. One of these reviews [32] discussed nine primary studies where group cohesion was reported as significantly associated with intention to stay. However, no detail was given about the nature of the interventions and variation in the reliability and validity of the measurement tools used was highlighted. Another review also described two studies demonstrating a positive impact of one year team oriented interventions (one of team discussion groups, the other undefined) on turnover [33]. Nursing practice models, for example nurse-managed units and unit-level self-management widely used by hospitals with Magnet accreditation in North America [43-45] were reported to have mixed evidence of effect [33].

### 3.8 Summary of Interventions and Their Effectiveness

Tables (**3** and **4**) present the interventions reported in the reviews, alongside a summary of evidence of effectiveness. In summary, the tables highlight that the specific or multiple interventions reviewed that may have an effect on retention or intention to stay are orientation programmes (including preceptorship, internships, residencies, and structured orientation programmes [34, 36] and mentorship [38]) for new graduates; transformational or relational leadership [32, 33]; and team work. [33] Retention is reported as highest when multiple interventions are used [33]. Clinical sabbaticals received some support although the review quality was low [37].

## 4 DISCUSSION

### 4.1 Summary of Findings From and Limitations of the Included Reviews

Seven reviews of interventions to reduce nurse turnover were found which had undertaken a quality appraisal of their included studies. These reviews provided consistent and important messages about what might work to increase retention or at least intention to stay. Firstly, they reported positive impact of transition programmes for newly qualified nurses. [34-36, 38]. These programmes were variously named, including the terms residency, internship and orientation as well as mentoring and preceptorship itself. Questions still remain as to the effectiveness, efficiency, and costs of different methods, frequency and duration of preceptorship programmes for newly qualified nurses who are in different types of clinical specialities and are themselves at a different life stages.

Secondly, the reviews also offered evidence of the positive impact of nurse manager leadership styles that were perceived as ‘transformative’ or encouragment of work group cohesion in reducing turnover or increasing retention (used interchangeably in this review) [32, 33]. Questions remain as to the extent these types of nurse leadership or management styles influence nurse turnover rates in different types of clinical services and in different types of organisational and job market contexts. In addition there are questions as to the effective (including judgements of cost) mechanisms for developing, maintaining and enacting these nurse leadership styles as judged by the primary outcome (rates of nurse turnover) and secondary outcomes such as described above.

Reviews of multiple interventions suggest that this approach is more effective than single interventions [33].

There was little overlap in the included primary studies in the reviews we analysed, due to their different foci, apart from two reviews of supportive programmes for newly graduated nurses [35, 36]. This finding highlights the importance of providing a summary of evidence from more than one systematic review on an important topic [19], as the separate reviews focus on different interventions or different sub-populations of adult nurses.

The quality of the reviews was in the AMSTAR category of moderate, although half of them were borderline with being classified as strong reviews. These included the quality appraisal of their included primary studies in weighting their discussion and drawing conclusions [32, 34, 35].

The reviews’ primary studies variously measured the intention to leave, turnover and retention, and the original reviews’ authors commented on the reduction in the strength of conclusions they could infer due to the weak study designs and variable validity and reliability of the measurement of these outcomes [32, 33]. Examples of weak designs commented on were the absence of control data in many of the primary studies [34, 36]. Other limitations noted by the original reviews’ authors included a predominantly North American focus [33, 35, 38], and a lack of focus on the retention of experienced nurses [33]. The absence of meta analysis in each review due to heterogeneity of studies was also a limitation [32].

In view of the critique of systematic reviews in terms of the ease of conducting poor quality ones that are given high esteem in publication and read by many who cannot easily differentiate their quality [46], we suggest that this summary has an important role to play in highlighting what is already known alongside where a lack of research studies leave space for uncertainty in the field of retention strategies for adult nursing.

### 4.2 Limitations and Strengths of Our Overview

Our overview is limited by design. Systematic reviews themselves are open to criticism as destructive of ‘reading, writing, thinking, interpreting, arguing and justifying [47]. As an overview (systematic review of systematic reviews) we have also relied upon the review authors’ reporting and interpretation of the primary studies and have made some assumptions about quality based on descriptions of research design if critical appraisal of each primary study was not clearly described in the reviews. We suggest that this limitation is mitigated by only including reviews that have reported a quality appraisal of their included studies. We have also assessed the quality of the included reviews using a widely recognised tool for this task [28]. We have therefore provided an account of what should be the highest quality reviews available although we note that there are no reviews which offer strong evidence, and the AMSTAR tool authors themselves promote further testing of the tool [28]. We have accepted review authors’ descriptions of heterogeneity as limiting opportunities for meta analysis without carrying out any formal analysis to consider whether the diversity has implications for the interpretation of findings. We have also treated the terms turnover and retention as direct opposites noting that the included reviews do not comment on their differences, although there are some authors who argue that though interrelated, they are conceptually different and the reduction of one does not necessarily lead to savings in the other in economic terms at least [48].

### 4.3 Our Findings in the Context of Other Literature

We initiated our investigation of the evidence on interventions to reduce adult nurse turnover following our early reading and subsequent interlinked systematic overview of the determinants of consequences of such turnover [8]. The finding we report there of a myriad of determinants of turnover, albeit a body of literature widely open to critique of its quality, which might suggest a myriad of linked interventions have been studied. In contrast, when we applied criteria based upon guidance for the good conduct of systematic reviews [15] we have found a fairly narrow range of interventions tested and reported. Moreover, interventions were tested with a narrow range of nurses, in studies which the reviews’ authors consider to lack rigour, particularly in the measurement of the primary outcome of interest, that is, the rate of turnover. In the context of the ongoing international phenomenon of nursing turnover and projected supply-demand concerns [6] this is a somewhat surprising finding.

It was also a surprising finding that otherwise well conducted reviews conflated evidence from self-reported determinants of turnover (*i.e.* what reports groups of nurses say are the causes of their intent or action to leave) with the review authors’ opinions as to strategies to decrease turnover (including in a very recent overview [49]). While the reviews we overviewed make it clear that there remains much to be done to improve the strength of evidence, for example there are no controlled trials and very few attempts to control observational studies, we argue such misapplied conflation has restricted the development of true intervention studies. In this we mean studies based on an intervention hypothesis for testing and framed by questions of interest to nurse and human resource managers which have the primary outcome of reduction of turnover rates (effectiveness) rates but secondary outcomes linked to accepted quality dimension criteria [50] such as of acceptability, patient safety and experience, staff well-being and cost consequences.

These findings are particularly pertinent when strategic guidance exists on good practice in staff retention [10, 51]. A comparison of the available guidance on retention of nursing in England and the evidence from our literature review [52] suggests there is some evidence that the current guidance offered to retain adult nurses is supported in part by the research literature regarding the determinants of turnover, [8] within the limitations of this evidence being of moderate strength. The guidance is supported in part by the research evidence on interventions with regard to developing nurse leaders and line managers, investing in the workforce and developing and continuing staff engagement [51] when we draw on the findings of two reviews [32, 33]. The strategy’s focus on newly qualified nurses is supported more widely supported by the evidence. [32, 33, 35, 36, 38] It would appear therefore that this is the most likely intervention to have a positive impact on retention. However this statement needs to be seen in the context that most of these studies were not true intervention studies and we are unable therefore to conclude that the strategies in the guidance would have the desired impact on turnover, according to the available research evidence.

## CONCLUSION

The current evidence on the effectiveness of interventions to reduce turnover in nursing workforces has a number of important limitations. However, it is important to note that a body of moderately high quality review evidence does exist giving a picture of a number of interventions – preceptorship of new graduates and leadership for group cohesion - that are evidenced to decrease turnover or increase retention. A management style by nurse managers that pays attention to a positive work environment and the nurse as an individual within that is also supported by the literature. However, large gaps remain in high quality evidence for interventions addressing the plethora of determinants of nurse turnover. While this is disappointing, the ongoing problems with retention and shortages of nurses in many countries mean that more research attention is required to build on the work reported here. We suggest that nurses and research funders should develop and test the interventions that were shown to be effective in observational or quasi-experimental studies in controlled studies, powered to allow for interrelated concepts on causal pathways to turnover, particularly with groups other than new graduate nurses, and designed with consistent primary and secondary outcome measures.

## SUPPLEMENTARY MATERIAL

Supplementary material is available on the publisher’s website along with the published article.

## Figures and Tables

**Fig. (1):**
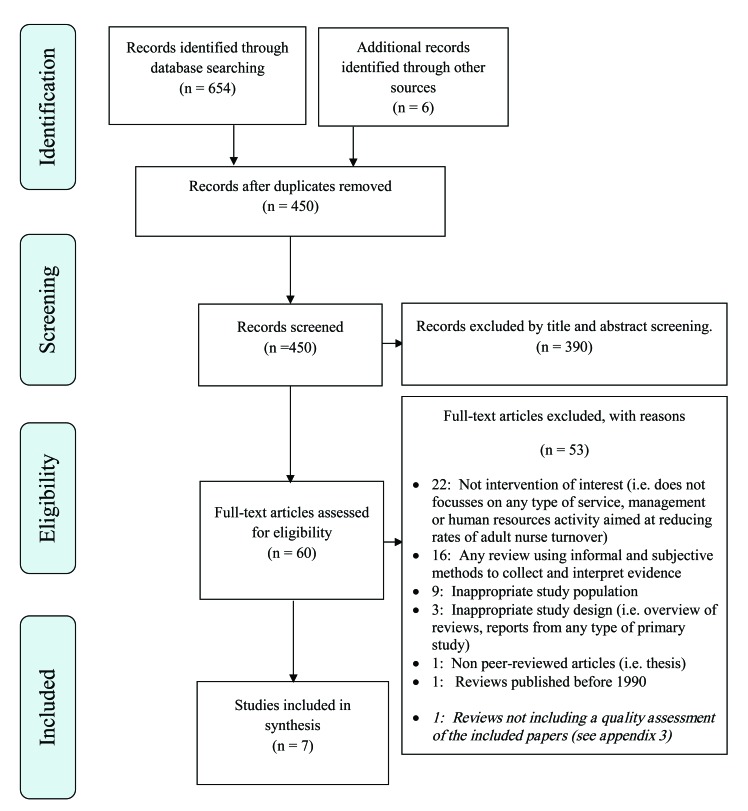
PRISMA flow diagram.

**Fig. (2):**
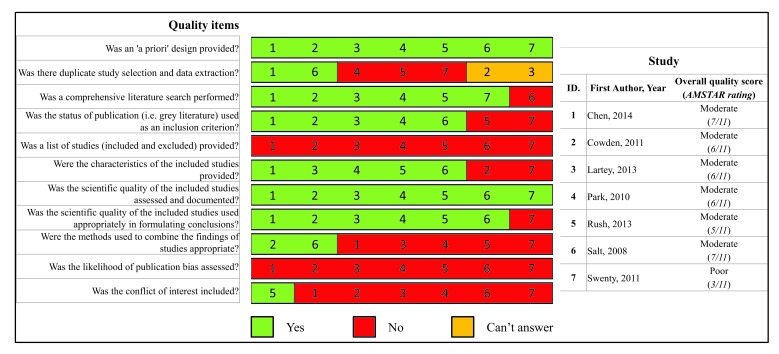
Graph of the methodological quality of the included reviews, according to AMSTAR quality items.

**Table 1:** Medline search strategy and number of articles found on 17/01/2015.

#	Searches	Results
1	exp Nursing Staff/	34106
2	exp Nursing Care/	58119
3	exp Nurses/	42050
4	(nurse or nurses or nursing).tw.	176170
5	1 or 2 or 3 or 4	229957
6	exp Personnel Turnover/	2974
7	((turnover adj3 (nurse or nurses or nursing)) or ((work or working or workload) adj3 (nurse or nurses or nursing)) or (leaving adj3 (nurse or nurses or nursing)) or (retention adj3 (nurse or nurses or nursing)) or (retain adj3 (nurse or nurses or nursing)) or (stay adj3 (nurse or nurses or nursing))).tw.	10391
8	6 or 7	12673
9	Job Satisfaction/ and (turnover* or leave or leaving or retention or retain or stay or staying).tw.	2220
10	Burnout/ and (turnover* or leave or leaving or retention or retain or stay or staying).tw.	644
11	Personnel Management/ and (turnover* or leave or leaving or retention or retain or stay or staying).tw.	379
12	Workload/ and (turnover* or leave or leaving or retention or retain or stay or staying).tw.	1018
13	((burnout or morale or stress) adj5 (turnover* or leave or leaving or retention or retain or stay or staying)).tw.	949
14	((economic* or financial or pay*) adj5 (turnover* or leave or leaving or retention or retain or stay or staying)).tw.	672
15	(job satisfaction adj5 (turnover* or leave or leaving or retention or retain or stay or staying)).tw.	421
16	((work or working or workload) adj5 (turnover* or leave or leaving or retention or retain or stay or staying)).tw.	2002
17	(organization* adj5 (turnover* or leave or leaving or retention or retain or stay or staying)).tw.	585
18	9 or 10 or 11 or 12 or 13 or 14 or 15 or 16 or 17	6758
19	8 or 18	17664
20	(incentive* or intervention* or strateg*).tw.	885703
21	(meta anal$ or metaanal$).ti,ab,sh.	77150
22	((methodol$ or systematic$ or quantitativ$) adj3 (review$ or overview$ or survey$)).ti,ab,sh.	63670
23	(medline or embase or index medicus).ti,ab.	57545
24	((pool$ or combined or combining) adj (data or trials or studies or results)).ti,ab.	10785
25	literature.ti,ab.	352373
26	21 or 22 or 23 or 24 or 25	456788
27	26 and review.pt,sh.	217357
28	5 and 19 and 20 and 27	176
29	limit 28 to english language	165

**Table 2:** General characteristics of the included systematic reviews.

**First Author** **year**	**Aim(s)** **Research question(s)**	**Selection criteria used to include primary studies (PICOS)**	**Scope** 1. Geography 2. Time limit 3. Language
Chen 2014^a^	To examine current information and clinical applications of mentorship programmes to attain a superior understanding of the implementation and effectiveness of such programmes for recently registered nurses.	*P* Recently RN *I* 1:1 Mentorship programme *C* No comparison groups *O* Retention; Turnover; Cost *S* Experimental or a quasi-experimental peer-reviewed primary studies	1 USA, ASIA 2 1999-2011 3 Not stated
Cowden 2011^b^	To examine the relationship between managers leadership practices and staff nurses intent to stay in their current position.	*P* Staff nurses *I* leadership practices *C* No comparison groups *O* Intention to stay *S* Peer-reviewed qualitative or quantitative studies	1 International (by Canada) 2 1985 - 2010 3 English
Larty 2014^c^	To report the effectiveness of strategies for retaining experienced RNs.	*P* Experienced RN’s (newly qualified excluded) *I* Any intervention aimed to increase the retention of experienced RNs *C* No comparison groups *O* Retention/ turnover *S* Quantitative research studies	1 Mostly USA 2 No limits 3 No limits
Park 2010^d^	To present an integrative review of the research that was conducted to explore the effects of orientation programs for newly graduated nurses on their confidence, competency, and retention.	*P* Hospital based, NGNs *I* Orientation programs in nursing literature (included internships, residencies, and structured orientation programs) *C* No comparison groups *O* Retention *S* Not stated	1 USA 2 1990-2007 3 English
Rush 2013^e^	The purpose of the study was to review existing research literature to identify best practices of formal new graduate nurse transition programs.	*P* NGNs within one year of graduation (acute care settings) *I* transition programs or orientation programs *C* No comparison groups *O* Retention; Turnover Cost-benefit *S* Any empirical study	1 Mostly USA 2 2000-2011 3 English
Salt 2008^f^	To conduct a systematic review of published research to determine the effectiveness of retention strategies targeted at NGNs.	*P* NGNs *I* A retention strategy was identified as a way to engage NGNs to continue service within a unit, hospital, or organization *C* No comparison groups *O* Retention *S* Only published and peer-reviewed primary studies	**1** USA **2** No limits **3** No limits
Swenty 2011^g^	To review and examine the literature supporting a professional sabbatical, a potentially viable and innovative change strategy that could renew, revitalize, and retain nursing staff practicing in the acute care setting. What is the evidence related to professional sabbaticals in nursing?	*P* Nursing, business & education *I* Clinical practice sabbatical *C* No comparison groups *O* Retention; Turnover *S* Not stated	1 USA 2 1999 - 2010 3 English

a [38], b [32], c [33], d [36], e [34], f [35], g [37],

**Table 3:** Summary of reviews presenting interventions with multiple strands.

**Intervention type**	**Effect** *Review authors’* *summary of findings*	**Supporting evidence**		
		**Type, number, and quality of included studies as reported by the review author(s)**	**Review quality score**	**Review reference** *First Author* *Year*
- Nursing practice models - Teamwork approach - Leadership practice - Organisational strategies - Individual strategies.	Most studies reported improved retention as a result of the intervention. Team work and individually targeted strategies including mentoring, leadership interest and in depth orientation increased job satisfaction and produced higher retention results. Retention was highest when multiple interventions were used.	**Total number 12** Quantitative 12 *Experimental (quasi) 2* *Observational 10*- *Quality* Quality assessment tool adapted from Estabrooks et al (2003)^a^ “All included studies were rated as medium or high in the quality assessment.” ^b^, page 1030	Moderate (*6/11*)	Larty 2014^b^
- Preceptor programme - Needs-based orientation programme - Residency programme - New graduate internship programme - Externship before graduation from a basic RN programme	Based on the strongest evidence, the highest retention rates were associated with retention strategies that used a preceptor programme model that focused on the NGN as well as a programme length of 3 to 6 months.	**Total number 16** Quantitative 16^^ *^^Type of quantitative studies included not discussed* Quality Quality assessment tool adapted from several existing frameworks (Cummings and Estabrooks 2003^c^, Estabrooks et al 2001^d^); “Eleven studies in the review were considered moderate, 3 were high, and 2 were weak.”^e,^ page 288	Moderate (*7/11*)	Salt 2008^e^

a [53], b [33], c [54], d [55], e [35]

**Table 4:** Articles most frequently included in the reviews assessed.

Articles		Salt	Park	Cowden	Swenty	Lartey	Rush	Chen
		2008 ^a [35]^	2010 ^b [36]^	2011 ^c [32]^	2011 ^d [37]^	2013 ^e [33]^	2013 ^f [34]^	2014 ^g [38]^
Owens ^h [61]^	2001	x	x					
Beecroft ^i [62]^	2001	x	x					
Squires ^j [63]^	2002	x	x					
Crimlisk ^k [64]^	2002	x	x					
Roche ^l [65]^	2004	x					x	
Almada ^m [39]^	2004	x	x				x	
Blanzola ^n [66]^	2004		x				x	
Marcum ^o [40]^	2004	x	x				x	
Altier ^p [41]^	2006	x	x				x	
Herdrich ^q [67]^	2006	x	x					
Keller ^r [68]^	2006		x				x	
Krugman ^s [42]^	2006	x	x				x	
Lee ^t [69]^	2009						x	x
Komaratat ^u [70]^	2009						x	x

a [35], b [36], c [32], d [37], e [33], f [34], g [38], h [61], i [62], j [63], k [64], l [65], m [39], n [66], o [40], p [41], q [67], r [68], s [42], t [69], u [70].

**Table 5:** Summary of reviews presenting specific interventions.

**Intervention type**	**Effect** *Review authors’* *summary of findings*	**Supporting evidence**		
		**Type, number, and quality of included studies as reported by the review’ author(s)**	**Review quality score**	**Review reference** *First Author* *Year*
Orientation programs	Orientation programmes (included internships, residencies, and structured orientation programmes) may encourage new graduates to stay in their current position	**Total number 17^** *^ Type of studies included not discussed* *Quality* Quality assessment tool adapted from Beck (2001) ^a^ No details available	Moderate (*6/11*)	Park 2010 ^b^
	The presence of a formal new graduate transition programme (or orientation programme) resulted in good retention of NGN and improved competency.	**Total number 47** Quantitative 15 *Experimental (quasi) 8* *Observational 7* Qualitative 5 Other* 27 * descriptive studies *Quality* Quality index with 3 criteria developed by Beck (2001)^a^ and later modified by Park and Jones (2010) ^b^ “Evidence was variable, and overall of low quality, limiting best practices recommendations.” ^c^	Moderate (*5/11*)	Rush 2013 ^c^
Mentorship Programmes	Mentorship programmes are a beneficial process for mentors and recently registered nurses. Results have shown that mentorship programmes improve competence, job satisfaction and reduce the turnover rate among recently registered nurses.	**Total number 5** Quantitative 5 *Experimental (quasi) 5* *Quality* Newman and Roberts (2002) ^d^ “significant reliability” ^e^ page 468	Moderate *(7/11)*	Chen 2014 ^e^
Leadership Practices	Managers’ leadership practices, Transformational or relational leadership approaches resulted in greater intentions to stay in their current positions. Other factors including perceived manager power, supervisor support, empowerment, involving them in decision making, and promotion of group cohesion all showed a significant positive correlation affecting the staff nurses intent to stay.	**Total number 23** Quantitative 22 *Experimental (quasi) -* *Observational 22* Qualitative - Mix-Methods 1 Other - *Quality* Quality assessment tool adapted from several existing frameworks (Cummings and Estabrooks 2003 ^f^, Wong and Cummings 2007 ^g^, Lee and Cummings 2008 ^h^); “..All studies were rated as moderate or strong” ^i^ page 468.	Moderate (*6/11*)	Cowden 2011 ^i^
Clinical practice sabbatical	The authors identified a nursing sabbatical as a viable option, which can enhance nursing retention and revitalization to generate positive outcomes.	**Total number 19** Quantitative 2 *Experimental (quasi) -* *Observational 2* Qualitative 3 Mix-Methods - Other 14** **opinion/ consensus papers *Quality* Stillwell, Fineout-Overholt, Melnyk and Williamson (2010) ^j^ Weak evidence^k^ page 157	Moderate (*3/11*)	Swenty 2011^k^

a [56], b [36], c [34], d [57], e [38], f [54], g [58], h [59], i [32], j [60], k [37], a [35], b [36], c [32], d [37], e [33], f [34], g [38], h [61], i [62], j [63], k [64], l [65], m [39], n [66], o [40], p [41], q [67], r [68], s [42], t [69], u [70],

## LIST OF ABBREVIATIONS

AMSTAR: = Assessment of Multiple Systematic Reviews
PICOS: = Population, Intervention, Comparison, Outcomes, Study design; P: Population; I: Intervention; C: Comparison; O: Outcome; S: Study design
PRISMA-P: = Preferred Reporting Items for Systematic review and Meta-analysis Protocols
NGN: = Newly Graduated Nurses;
RN: = Registered Nurse
