# Supplementary material for: Interventions to Reduce Adult Nursing Turnover: A Systematic Review of Systematic Reviews
Source: Open Nurs J. 2017 Aug 15;11:108–23. doi: 10.2174/1874434601711010108 (PMC5725565; doi:10.2174/1874434601711010108)
Supplement: Supplementary file 1 [file TONURSJ-11-108_SD1.pdf]

Send Orders for Reprints to reprints@benthamscience.ae

BENTHAM OPEN

CrossMark

The Open Nursing Journal

Supplementary Material

Content list available at: www.benthamopen.com/TONURSJ/

DOI: 10.2174/1874434601711010108

Interventions to Reduce Adult Nursing Turnover: A Systematic Review of Systematic Reviews

Mary Halter[1,*], Ferruccio Pelone[2], Olga Boiko[1], Carole Beighton[1], Ruth Harris[3], Julia Gale[1], Stephen Gourlay[4] and Vari Drennan[1]

[1]*Faculty of Health, Social Care and Education, Kingston University and St George's, University of London, London, England*
[2]*National Guideline Alliance, Royal College of Obstetricians and Gynaecologists, London, England*
[3]*Nursing & Midwifery, King's College London, England*
[4]*Faculty of Business & Law, Kingston University, London, England*

Received: March 20, 2017 Revised: May 22, 2017 Accepted: June 08, 2017

SUPPLEMENTARY FILE 1

Adult Nurse Turnover: OVERVIEW Search Strategies for Individual Databases on Interventions

Supplementary Table 1. EMBASE (OVID): Database inception-2015, searched on February 2015.

#	Searches	Results
1	exp Nursing Staff/	37048
2	exp Nursing Care/	11887
3	exp Nurses/	88351
4	(nurse or nurses or nursing).tw.	232423
5	1 or 2 or 3 or 4	273819
6	exp Personnel Turnover/	45244
7	(turnover or (leave adj3 (nurse or nurses or nursing)) or (leaving adj3 (nurse or nurses or nursing)) or (retention adj3 (nurse or nurses or nursing)) or (retain adj3 (nurse or nurses or nursing)) or (stay adj3 (nurse or nurses or nursing))).tw.	57080
8	6 or 7	100754
9	Personnel Management/ and (incentive* or intervention* or strateg*).tw.	4235
10	workload/ and (incentive* or intervention* or strateg*).tw.	3293
11	Burnout, Professional/ and (incentive* or intervention* or strateg*).tw.	1563
12	Job Satisfaction/ and (incentive* or intervention* or strateg*).tw.	2328
13	((burnout or stress) adj3 (incentive* or intervention* or strateg*)).tw.	3027
14	((work or working or workload) adj3 (incentive* or intervention* or strateg*)).tw.	4731
15	(organi?ation* adj3 (incentive* or intervention* or strateg*)).tw.	2972
16	((economic* or financial or pay*) adj3 (incentive* or intervention* or strateg*)).tw.	6640
17	9 or 10 or 11 or 12 or 13 or 14 or 15 or 16	25594
18	5 and 8 and 17	1817
19	exp nursing staff/	37048
20	exp nursing care/	11887
21	exp nurse/	88351
22	(nurse or nurses or nursing).tw.	232423
23	19 or 20 or 21 or 22	273819
24	Personnel Turnover.mp.	46

Suppl. Table 1 contd.....

#	Searches	Results
25	*personnel management/ or exp health care personnel management/ or exp personnel shortage/	20535
26	((turnover adj5 (nurse or nurses or nursing)) or (leave adj5 (nurse or nurses or nursing)) or (leaving adj5 (nurse or nurses or nursing)) or (retention adj5 (nurse or nurses or nursing)) or (retain adj5 (nurse or nurses or nursing)) or (stay adj5 (nurse or nurses or nursing))).tw.	3045
27	Job Satisfaction/ and (turnover* or leave or leaving or retention or retain or stay or staying).tw.	2568
28	Burnout/ and (turnover* or leave or leaving or retention or retain or stay or staying).tw.	770
29	Personnel Management/ and (turnover* or leave or leaving or retention or retain or stay or staying).tw.	3226
30	Workload/ and (turnover* or leave or leaving or retention or retain or stay or staying).tw.	1486
31	((burnout or morale or stress) adj5 (turnover* or leave or leaving or retention or retain or stay or staying)).tw.	1280
32	((economic* or financial or pay*) adj5 (turnover* or leave or leaving or retention or retain or stay or staying)).tw.	1064
33	(job satisfaction adj5 (turnover* or leave or leaving or retention or retain or stay or staying)).tw.	517
34	((work or working or workload) adj5 (turnover* or leave or leaving or retention or retain or stay or staying)).tw.	2781
35	(organi?ation* adj5 (turnover* or leave or leaving or retention or retain or stay or staying)).tw.	715
36	24 or 25 or 26 or 27 or 28 or 29 or 30 or 31 or 32 or 33 or 34 or 35	30348
37	Meta-Analysis as Topic/	17217
38	meta analy$.tw.	86493
39	metaanaly$.tw.	4528
40	Meta-Analysis/	83196
41	(systematic adj (review$1 or overview$1)).tw.	73971
42	exp Review Literature as Topic/	24995
43	cochrane.ab.	40451
44	embase.ab.	39177
45	(psychlit or psyclit).ab.	928
46	(psychinfo or psycinfo).ab.	9560
47	(cinahl or cinhal).ab.	12351
48	science citation index.ab.	2171
49	bids.ab.	384
50	cancerlit.ab.	635
51	37 or 38 or 39 or 40 or 41 or 42	197027
52	43 or 44 or 45 or 46 or 47 or 48 or 49 or 50	63878
53	reference list$.ab.	10908
54	bibliograph$.ab.	12740
55	hand-search$.ab.	4842
56	relevant journals.ab.	848
57	manual search$.ab.	2831
58	53 or 54 or 55 or 56 or 57	28725
59	selection criteria.ab.	18764
60	data extraction.ab.	11832
61	59 or 60	29444
62	Review/	1545292
63	61 and 62	15706
64	Comment/	0
65	Letter/	573045
66	Editorial/	373456
67	64 or 65 or 66	946333
68	51 or 52 or 58 or 63	230111
69	68 not 67	219913
70	23 and 36 and 69	190
71	limit 70 to english language	185
72	(incentive* or intervention* or strateg*).tw.	1318166
73	71 and 72	79
74	limit 73 to english language	79

The Cochrane Database of Systematic Reviews: database inception-2015, searched February 2015.

#	Searches	Results
1	intervention:ti,ab,kw (Word variations have been searched)	97723
2	incentive*:ti,ab,kw (Word variations have been searched)	1418
3	strateg*:ti,ab,kw (Word variations have been searched)	29745
4	#1 or #2 or #3	117754
5	MeSH descriptor: [Nursing Care] explode all trees	1615
6	MeSH descriptor: [Nursing Staff] explode all trees	529
7	nurse:ti,ab,kw or "Nurses'":ti,ab,kw or "nursing":ti,ab,kw (Word variations have been searched)	16270
8	#5 or #6 or #7	16294
9	MeSH descriptor: [Personnel Turnover] explode all trees	20
10	MeSH descriptor: [Burnout, Professional] explode all trees	134
11	MeSH descriptor: [Workload] explode all trees	321
12	MeSH descriptor: [Job Satisfaction] explode all trees	175
13	retain:ti,ab,kw or "retention":ti,ab,kw or "leave":ti,ab,kw or "stay":ti,ab,kw or "staying":ti,ab,kw (Word variations have been searched)	52436
14	#13 and (#9 or #10 or #11 or #12)	78
15	#14 and #8 and 3	5

CINAHL (EBSCO): Database inception-2015, searched February 2015.

#	Searches	Results
1	(MM "Staff Nurses")	3 627
2	(MH "Nursing Care+")	244 145
3	(MH "Nurses+")	166 259
4	(MH "Adult Nurse Practitioners")	83
5	TX (nurse or nurses or nursing)	1 198 126
6	S1 OR S2 OR S3 OR S4 OR S5	1 203 824
7	TX (turnover N5 (nurse or nurses or nursing)) or (leave N5 (nurse or nurses or nursing)) or (leaving N5 (nurse or nurses or nursing)) or (retention N5 (nurse or nurses or nursing)) or (retain N5 (nurse or nurses or nursing)) or (stay N5 (nurse or nurses or nursing)))	5 182
8	(MH "Personnel Turnover") AND TX ((nurse or nurses or nursing))	2 182
9	(MH "Job Satisfaction") AND TX (turnover* or leave or leaving or retention or retain or stay or staying))	3 269
10	(MH "Burnout, Professional") AND TX (turnover* or leave or leaving or retention or retain or stay or staying))	649
11	(MH "Workload") AND TX (turnover* or leave or leaving or retention or retain or stay or staying))	959
12	TX ((burnout or morale or stress) N5 (turnover* or leave or leaving or retention or retain or stay or staying))	637
13	TX ((economic* or financial or pay*) N5 (turnover* or leave or leaving or retention or retain or stay or staying))	1 863
14	TX ("job satisfaction" N5 (turnover* or leave or leaving or retention or retain or stay or staying))	727
15	TX ((work or working or workload) N5 (turnover* or leave or leaving or retention or retain or stay or staying))	1 839
16	TX (organi?ation* N5 (turnover* or leave or leaving or retention or retain or stay or staying))	726
17	S7 OR S8 OR S9 OR S10 OR S11 OR S12 OR S13 OR S14 OR S15 OR S16	12 404
18	TX (incentive* or intervention* or strateg*)	341 467
19	(MH "Meta Analysis")	21 278
20	(MH "Systematic Review")	29 650
21	PT systematic review	47 197
22	TX (metaanaly* or meta-analy*)	31 579
23	TX metanal*	26
24	PT nursing interventions	1 426
25	TI (review* or overview*)	120 525
26	(MH "Literature Review")	3 670
27	(MH "Literature Searching+")	6 960
28	TX cochrane*	23 515
29	TX synthes* N3 (literature* or research* or studies or data)	5 222
30	TX (MEDLINE or medlars or embase or scisearch or psycinfo or psychinfo or psyclit or psychlit)	43 197
31	TX pooled analy*	2 523
32	TX ((data N2 pool*) and studies)	1 583

Contd.....

#	Searches	Results
33	TX ((hand or manual* or database* or computer*) N2 search*)	15 114
34	(MH “Reference Databases”)	2 104
35	TX ((electronic* or bibliographic*) N2 (database* or data base*))	4 960
36	PT (review or systematic-review or practice-guidelines)	117 088
37	AB (review* or overview*)	194 695
38	AB (systematic* or methodologic* or quantitativ* or research* or literature* or studies or trial* or effective*)	563 840
39	S36 AND S38	48 949
40	S36 N10 S38	48 949
41	S18 OR S19 OR S20 OR S21 OR S22 OR S23 OR S24 OR S25 OR S26 OR S27 OR S28 OR S29 OR S30 OR S31 OR S32 OR S33 OR S34 OR S35 OR S39 OR S40	522 176
42	PT editorial	194 451
43	PT letter	195 566
44	PT case study	263 832
45	(MH “record review”)	42 948
46	(MH “peer review”)	4 348
47	TX (retrospective* N2 review*)	13 968
48	TX (case* N2 review*)	10 785
49	TX (record* N2 review*)	46 459
50	TX (patient* N2 review*)	10 343
51	TX (patient* N2 chart*)	2 542
52	TX (peer N2 review*)	3 244 495
53	TX (chart* N2 review*)	7 200
54	TX (case* N2 report*)	51 857
55	(MH “Case Control Studies+”)	50 075
56	(MH “Prospective Studies+”)	254 352
57	(MH “case studies)	15 088
58	(MH “animal studies)	63 286
59	(MH “edit and review”)	1 891
60	TX (rat* or mouse or mice or hamster* or animal* or dog* or cat* or rabbit* or bovine or sheep)	704 989
61	S42 OR S43 OR S44 OR S45 OR S46 OR S47 OR S48 OR S49 OR S50 OR S51 OR S52 OR S53 OR S54 OR S55 OR S56 OR S57 OR S58 OR S59 OR S60	3 492 972
62	S19 OR S20 OR S21 OR S22 OR S23 OR S24 OR S25 OR S26 OR S27 OR S28 OR S29 OR S30 OR S31 OR S32 OR S33 OR S34 OR S35 OR S39 OR S40	222 100
63	S61 NOT (S61 AND S62)	3 292 263
64	S62 NOT S63	222 100
65	S6 AND S17 AND S18 AND S64 Limiters - Language: English	156

Applied Social Sciences Index and Abstracts –ASSIA (ProQuest XML) – Database inception-2015, searched February 2015.

#	Searches	Results
S5	S4 and S3 AND S2 AND S1	51
S4	(literature review) OR ti((review OR meta-analysis)) OR ab(review) OR (SU.EXACT(“Systematic reviews”) OR SU.EXACT(“Reviews”))	52501
S3	(all(incentive*) or all(intervention*) or all(strateg*))	101714
S2	((all(leave) NEAR/5 (all(nurse) OR all(nurses) OR all(nursing))) OR (all(leaving) NEAR/5 (all(nurse) or all(nurses) or all(nursing)) OR all(retention) NEAR/5 (all(nurse) or all(nurses) or all(nursing))) OR SU.EXACT.EXPLODE(“Turnover”)) OR (SU.EXACT.EXPLODE(“Burnout”) AND (turnover* OR leave OR leaving OR retention OR retain OR stay OR staying)) OR ((turnover* OR leave OR leaving OR retention OR retain OR stay OR staying) AND SU.EXACT.EXPLODE(“Job applicants” OR “Job redesign” OR “Personnel management”)) OR ((turnover* OR leave OR leaving OR retention OR retain OR stay OR staying) AND SU.EXACT.EXPLODE(“Workloads”)) OR ((all(burnout) or all(morale) or all(stress)) NEAR/5 (all(turnover*) or all(leave) or all(leaving) or all(retention) or all(retain) or all(stay) or all(staying))) OR ((all(economic*) or all(financial) or all(pay*)) NEAR/5 (all(turnover*) or all(leave) or all(leaving) or all(retention) or all(retain) or all(stay) or all(staying))) OR ((work or working or workload) NEAR/5 (turnover* or leave or leaving or retention or retain or stay or staying))	2331
S1	nurse OR (nurses OR nursing) OR SU.EXACT(“Nursing”) OR (SU.EXACT(“Staff nurses”) OR SU.EXACT(“Nurses”))	66598

Scopus: database inception-2015, searched February 2015

#	Searches	Results
1	TITLE-ABS-KEY (“nurse” or “nurses” or “nursing”)	393865
2	TITLE-ABS (“turnover” W/5 “nurse” or “nurses” or “nursing”)	1262
3	TITLE-ABS (“leave” W/5 “nurse” or “nurses” or “nursing”)	978
4	TITLE-ABS (“leaving” W/5 “nurse” or “nurses” or “nursing”)	507
5	TITLE-ABS (“retention” W/5 “nurse” or “nurses” or “nursing”)	2292
6	TITLE-ABS (“retain” W/5 “nurse” or “nurses” or “nursing”)	616
7	TITLE-ABS (“staying” W/5 “nurse” or “nurses” or “nursing”)	173
8	TITLE-ABS (“stay” W/5”nurse” or “nurses” or “nursing”)	3267
10	TITLE-ABS (“work” W/3 “turnover” or “leave” or “leaving” or “retention” or “retain” or “stay” or “staying”)	5623
11	TITLE-ABS (“organi?ation” W/5 “turnover” or “leave” or “leaving” or “retention” or “retain” or “stay” or “staying”)	3576
12	TITLE-ABS (“organi?ational” W/5 “turnover” or “leave” or “leaving” or “retention” or “retain” or “stay” or “staying”)	2287
13	TITLE-ABS (“pay” W/10 “turnover” or “leave” or “leaving” or “retention” or “retain” or “stay” or “staying”)	890
14	TITLE-ABS (“financial” W/10 “turnover” or “leave” or “leaving” or “retention” or “retain” or “stay” or “staying”)	1568
15	TITLE-ABS (“stress” W/10 “turnover” or “leave” or “leaving” or “retention” or “retain” or “stay” or “staying”)	4564
16	TITLE-ABS (“Workload” W/10 “turnover” or “leave” or “leaving” or “retention” or “retain” or “stay” or “staying”)	412
17	TITLE-ABS (“Burnout” W/10 “turnover” or “leave” or “leaving” or “retention” or “retain” or “stay” or “staying”)	546
18	TITLE-ABS (“Job Satisfaction” W/10 “turnover” or “leave” or “leaving” or “retention” or “retain” or “stay” or “staying”)	1292
19	or/2-18	18823
20	TITLE-ABS-KEY (“literature review”)	94620
21	TITLE-ABS (“review” OR “meta-analysis”)	1874148
22	TITLE-ABS-KEY (“Systematic review”)	11,284
23	or/20-22	1989869
24	TITLE-ABS (“incentive” OR “incentives” OR “intervention” OR “interventions” OR “strategy” OR “strategies”)	2182551
25	1 AND 19 AND 23 AND 24	210

SUPPLEMENTARY FILE 2

Adult Nurse Turnover OVERVIEW THEMATIC INDEX_Interventions

Index of Interventions

1. Individual level

Population: the review was focused on those delivering adult nursing (*i.e.* licensed or registered) in health services (both in hospital and community) in developed economies (according to the definition of the International Monetary Fund [25]);

1.1. One to one mentorship programmes
1.2. Individual strategies
1.3. Internships, externships
1.4. Transition to speciality practice programs
1.5. (Partnership) orientation programs
1.6. Bicultural training

2. Leadership Level

2.1. Leadership/management training
2.2. Supervisor support
2.3. Preceptor program

3. Group Level

3.1. Residency programmes
3.2. Group cohesion
3.3. Peer support

3.4. Nursing practice models
3.5. Team-oriented interventions

4. Organisational Level
4.1. Family friendly policies

SUPPLEMENTARY FILE 3

Adult Nurse Turnover OVERVIEW Excluded Studies_Interventions

Part 1: Full-text articles excluded, with reasons.

Part 2: General characteristics of the excluded *i.e.* no literature review: *e.g.* overview of reviews, reports from any type of primary study reviews (those not including a quality assessment of the included papers).

Part 1: Full-text articles excluded, with reasons.

Reason for exclusion	References of excluded studies
**Not intervention of interest**	1. Anderson G, Hair C, Todero C. Nurse Residency Programs: An Evidence-Based Review of Theory, Process, and Outcomes. Journal of Professional Nursing. 2012;28(4):203-12.<br>2. Attree M, Flinkman M, Howley B, Lakanmaa R-, Lima-Basto M, Uhrenfeldt L. A review of nursing workforce policies in five European countries: Denmark, Finland, Ireland, Portugal and United Kingdom/England. J Nurs Manag. 2011.<br>3. Barlow KM, Zangaro GA. Meta-analysis of the reliability and validity of the Anticipated Turnover Scale across studies of registered nurses in the United States. J Nurs Manag. 2010;18(7):862-73.<br>4. Bott M. Research review. A mentorship program. CANNT Journal 2005;<br>5. Buchan J. Nursing shortages and evidence-based interventions: A case study from Scotland. Int Nurs Rev. 2002;49(4):209-18.<br>6. Butler M, Collins R, Drennan J, Halligan P, O'Mathúna DP, Schultz TJ, *et al.* Hospital nurse staffing models and patient and staff-related outcomes. Cochrane database of systematic reviews (Online). 2011(7).<br>7. Chan CW, Perry L. Lifestyle health promotion interventions for the nursing workforce: A systematic review. J Clin Nurs. 2012;21(15-16):2247-61.<br>8. Chang EM, Hancock KM, Johnson A, Daly J, Jackson D. Role stress in nurses: Review of related factors and strategies for moving forward. Nursing and Health Sciences. 2005;7(1):57-65.<br>9. Coomber B, Louise Barriball K. Impact of job satisfaction components on intent to leave and turnover for hospital-based nurses: A review of the research literature. Int J Nurs Stud. 2007;44(2):297-314.<br>10. Crenshaw JT. Use of video-feedback, reflection, and interactive analysis to improve nurse leadership Practices. Nurs Adm Q. 2012;36(3):260-7.<br>11. Edward KL, Hercelinskyj G. Burnout in the caring nurse: learning resilient behaviours. Br J Nurs. 2007;16(4):240-2.<br>12. Flint A, Webster J. Exit interviews to reduce turnover amongst healthcare professionals. The Cochrane database of systematic reviews. 2013;3.<br>13. Frehywot S, Mullan F, Payne PW, Ross H. Compulsory service programmes for recruiting health workers in remote and rural areas: do they work? Bull World Health Organ. 2010 May;88(5):364-70. doi: 10.2471/BLT.09.071605.<br>14. Gazza EA, Hunker DF. Facilitating student retention in online graduate nursing education programs: A review of the literature. Nurse Educ Today. 2014;34(7):1125-9.<br>15. Gilbody S, Cahill J, Barkham M, Richards D, Bee P, Glanville J. Can we improve the morale of staff working in psychiatric units? A systematic review. Journal of Mental Health. 2006;15(1):7-17.<br>16. Gilliss CL, Powell DL, Carter B. Recruiting and retaining a diverse workforce in nursing: From evidence to best practices to policy. Policy, Politics, and Nursing Practice. 2010;11(4):294-301.<br>17. Gonzalez R. Pietsch TT. Kozub K. Cole P. Nifras R. Russell-Headley K. Durhams T. Tomesko J. Wondolowski G. Caring: looking beyond simulations. International Journal for Human Caring 2010;<br>18. Hildebrandt L. Providing grief resolution as an Oncology nurse retention strategy: A literature review. Clin J Oncol Nurs. 2012;16(6):601-6.<br>19. Lobo VM. Transformational leadership to promote cross-generational retention. Nursing leadership (Toronto, Ont.). 2010;23(2):21-31.<br>20. Nelsey L, Brownie S. Effective leadership, teamwork and mentoring - Essential elements in promoting generational cohesion in the nursing workforce and retaining nurses. Collegian. 2012;19(4):197-202.<br>21. Twigg D, McCullough K. Nurse retention: A review of strategies to create and enhance positive practice environments in clinical settings. Int J Nurs Stud. 2014;51(1):85-92.<br>22. Webster J, Flint A. Exit interviews to reduce turnover amongst healthcare professionals. The Cochrane database of systematic reviews. 2014;8.

Part 1 contd.....

Reason for exclusion	References of excluded studies
**Any review using informal and subjective methods to collect and interpret evidence**	1. AL-Dossary R, Kitsantas P, Maddox PJ. The impact of residency programs on new nurse graduates' clinical decision-making and leadership skills: A systematic review. Nurse Educ Today. 2014;34(6):1024-8.<br>2. Becker DM. Implementing a night-shift clinical nurse specialist. Clinical Nurse Specialist. 2013;27(1):26-30.<br>3. Cummins A. Clinical supervision: The way forward? A review of the literature. Nurse Education in Practice. 2009;9(3):215-20.<br>4. Force MV. The relationship between effective nurse managers and nursing retention. J Nurs Adm. 2005;35(7-8):336-41.<br>5. Hutchinson D, Brown J, Longworth K. Attracting and maintaining the Y Generation in nursing: A literature review. J Nurs Manag. 2012;20(4):444-50.<br>6. Jackson D, Mannix J, Daly J. Retaining a viable workforce: a critical challenge for nursing. Contemporary nurse: a journal for the Australian nursing profession. 2001;11(2-3):163-72.<br>7. Kleinman CS. Leadership: a key strategy in staff nurse retention. J Contin Educ Nurs. 2004;35(3):128-32.<br>8. McDonald AW, Ward-Smith P. A review of evidence-based strategies to retain graduate nurses in the profession. Journal for Nurses in Staff Development. 2012;28(1):E16-20.<br>9. Mills JE, Francis KL, Bonner A. Mentoring, clinical supervision and preceptoring: clarifying the conceptual definitions for Australian rural nurses. A review of the literature. Rural and remote health. 2005;5(3):410.<br>10. Moseley A, Jeffers L, Paterson J. The retention of the older nursing workforce: A literature review exploring factors that influence the retention and turnover of older nurses. Contemporary Nurse. 2008;30(1):46-56.<br>11. O'Leary KJ, Sehgal NL, Terrell G, Williams MV. Interdisciplinary teamwork in hospitals: A review and practical recommendations for improvement. Journal of Hospital Medicine. 2012;7(1):48-54.<br>12. Smith-Stoner M, Markley J. Home healthcare nurse recruitment and retention: Tips for retaining nurses: One state's experience. Home Healthc Nurse. 2007;25(3):198-205.<br>13. Stichler JF. Healthy work environments for the ageing nursing workforce. J Nurs Manag. 2013;21(7):956-63.<br>14. Storey C, Cheater F, Ford J, Leese B. Retention of nurses in the primary and community care workforce after the age of 50 years: Database analysis and literature review. J Adv Nurs. 2009;65(8):1596-605.<br>15. Whitehead B, Owen P, Holmes D, Beddingham E, Simmons M, Henshaw L, *et al.* Supporting newly qualified nurses in the UK: A systematic literature review. Nurse Educ Today. 2013;33(4):370-7.<br>16. Willemsen-McBride T. Preceptorship planning is essential to perioperative nursing retention: matching teaching and learning styles. Can Oper Room Nurs J. 2010;28(1):8, 10,"11, 16 passim".
**Inappropriate study population**	1. Bärnighausen T, Bloom DE. Financial incentives for return of service in underserved areas: A systematic review. BMC Health Services Research. 2009;9.<br>2. Batcheller J. Chief Nursing Officer Turnover: An Analysis of the Literature. Nurs Clin North Am. 2010;45(1):11-31.<br>3. Brown P, Fraser K, Wong CA, Muise M, Cummings G. Factors influencing intentions to stay and retention of nurse managers: A systematic review. J Nurs Manag. 2013;21(3):459-72.<br>4. Buykx P, Humphreys J, Wakerman J, Pashen D. Systematic review of effective retention incentives for health workers in rural and remote areas: towards evidence-based policy. Aust J Rural Health. 2010;18(3):102-9.<br>5. Fisher KA, Fraser JD. Rural health career pathways: Research themes in recruitment and retention. Australian Health Review. 2010;34(3):292-6.<br>6. Flint A, Webster J. The use of the exit interview to reduce turnover amongst healthcare professionals. Cochrane database of systematic reviews (Online). 2011;1.<br>7. Gagnon MP, Pollender H, Trépanier A, Duplàa E, Ly BA. Supporting health professionals through information and communication technologies: a systematic review of the effects of information and communication technologies on recruitment and retention. Telemedicine journal and e-health: the official journal of the American Telemedicine Association. 2011;17(4):269-74.<br>8. Suter E, Deutschlander S, Mickelson G, Nurani Z, Lait J, Harrison L, *et al.* Can interprofessional collaboration provide health human resources solutions? A knowledge synthesis. Journal of Interprofessional Care. 2012;26(4):261-8.<br>9. Varekamp I, Verbeek JHAM, van Dijk FJH. How can we help employees with chronic diseases to stay at work? A review of interventions aimed at job retention and based on an empowerment perspective. Int Arch Occup Environ Health. 2006;80(2):87-97.
**Inappropriate study design (*i.e.* overview of reviews, reports from any type of primary study)**	1. Chenoweth L, Jeon YH, Merlyn T, Brodaty H. A systematic review of what factors attract and retain nurses in aged and dementia care. J Clin Nurs. 2010;19(1-2):156-67.<br>2. Mbemba G, Gagnon M-, Paré G, Côté J. Interventions for supporting nurse retention in rural and remote areas: An umbrella review. Human Resources for Health. 2013;11(1).<br>3. Tourangeau AE, Thomson H, Cummings G, Cranley LA. Generation-specific incentives and disincentives for nurses to remain employed in acute care hospitals. J Nurs Manag. 2013;21(3):473-82.
**Non peer-reviewed articles (*i.e.* thesis)**	1. Wilkes J, Epps. Best practices: Retaining registered nurses 2010, Dissertation
**Reviews published before 1990**	1. Helmer FT, MCknight P. Management strategies to minimize nursing turnover. Health Care Manage Rev. 1989;14(1):73-80.

Part 1 contd.....

Reason for exclusion	References of excluded studies
**Reviews not including a quality assessment of the included papers** ***(please see part 2 of this file for further details)***	1. Morphet J, Considine J, McKenna L. Transition to specialty practice programs in emergency nursing - A review of the literature. Australasian Emergency Nursing Journal. 2011; 14(1):45-9.

Part 2: General characteristics of the excluded reviews (those not including a quality assessment of the included papers).

First Author year	Aim(s) Research question(s)	Selection criteria used to include primary studies (PICOS)	Scope 1. Geography 2. Time limit 3. Language	Type, number, and quality of included studies as reported by the author(s)	Review authors' summary of findings	AMSTAR score
Morphet 2011	To present a review of the literature related to Transition to specialty practice programs (TSPPs) with a focus on emergency nursing	*P* Staff nurses<br>*I* Transition to specialty practice programs (TSPPs)<br>*C* Not stated<br>*O* Retention Attrition<br>*S* Not stated	1 International (by Australia)<br>2 1990 –2010<br>3 English	Total number* 11<br>Quantitative -<br>*Experimental (quasi)* -<br>*Observational* -<br>Qualitative -<br>Mix-Methods -<br>Other -<br>*No details available.	Transition to specialty practice programs (TSPPs) which offer elements of extended orientation, education and preceptorship have been successful in recruiting and short-term retention of nursing staff in emergency nursing.	Moderate risk of bias 5/11

PICOS: Population, Intervention, Comparison, Outcomes, Study design; P: Population; I: Intervention; C: Comparison; O: Outcome; S: Study design

© 2017 Halter *et al.*

This is an open access article distributed under the terms of the Creative Commons Attribution 4.0 International Public License (CC-BY 4.0), a copy of which is available at: https://creativecommons.org/licenses/by/4.0/legalcode. This license permits unrestricted use, distribution, and reproduction in any medium, provided the original author and source are credited.
